# Health risk stratification based on computed tomography pulmonary artery obstruction index for acute pulmonary embolism

**DOI:** 10.1038/s41598-018-36115-7

**Published:** 2018-12-17

**Authors:** Fei Guo, Guanghui Zhu, Junjie Shen, Yichuan Ma

**Affiliations:** Department of Radiology, The First Affiliated Hospital of Bengbu Medical College, Bengbu, 233004 China

## Abstract

Early effective identification of high-risk patients for acute pulmonary embolism (APE) contributes to timely treatment. The pulmonary artery obstruction index (PAOI) in computed tomography angiography (CTA) is a semi-quantitative observation index, commonly used to evaluate the severity of a patient’s condition. This study explores the ability of PAOI to assess the risk stratification of APE. Thirty patients with APE were analysed. They were classified according to the guidelines, and the PAOI and cardiovascular parameters were measured in CTA. The difference of PAOI between different risk stratification patients was compared, and the predictive value of the PAOI for high-risk stratification was evaluated by the receiver operating characteristic curve. The correlation between PAOI and cardiovascular parameters was also analysed by Spearman correlation analysis. The PAOI in low- and high-risk patients was (33.2 ± 18.6)% and (68.1 ± 11.8)% respectively, and the difference was statistically significant. The PAOI was strongly predictive for high-risk patients. The cut-off value was 52.5%, with a sensitivity of 100% and specificity of 81.0%. The PAOI was correlated with the main cardiovascular parameters. We conclude that the PAOI in CTA is helpful for assessing risk stratification in patients with APE, which contributes to the selection of both the treatment plan and prognostic evaluation.

## Introduction

Acute pulmonary embolism (APE) is a group of clinical and pathophysiological syndromes caused by embolism of an endogenous embolus (from coagulated blood, fat, amniotic fluid or tumour) or an exogenous embolus (such as gas embolus) in the pulmonary artery or its branches^1^. It has been reported that APE may cause many complications such as cardiogenic shock, sudden death, etc^2–5^. The mortality rate can be as high as 31–36% for such cases^6^. If early diagnosis and effective anticoagulant therapy can be taken in time, the mortality rate can be reduced to 2–10%^7^. However, due to the lack of specific clinical symptoms and signs, pulmonary embolism is easily misdiagnosed or missed, and the best opportunity for treatment may therefore be missed^8,9^.

An accurate understanding of the developmental process of pulmonary embolism, as well as accurate risk stratification for the patient’s condition, can assist in making an appropriate treatment plan; thereby improving the curative effect for the patient^10^. In 2008, the European Society of Cardiology (ESC) suggested that the area of the embolism should be replaced with risk stratification^11^. In 2014, the ESC updated the “Guidelines on the diagnosis and management of acute pulmonary embolism”^12^. The guidelines described that the risk stratification during diagnosis should be combined with consideration of right heart function and myocardial injury. A reliable risk stratification is the cornerstone of APE treatment^13,14^, However, the method for risk stratification involves numerous clinical indicators, and is still restricted with regard to its clinical application.

With the application of multi-slice spiral CT pulmonary angiography (CTPA), the accuracy of early diagnosis of APE is greatly improved^15,16^. In addition to the diagnosis and differential diagnosis, CTPA was also used to evaluate prognosis and so on. Based on CTPA, Qanadli *et al*. established a semi-quantitative analysis method for APE, called the pulmonary artery obstruction index (PAOI)^17^. It is reported that the PAOI was related to the severity of APE^18^. However, very little of the literature has focused on the correlation between PAOI and the risk stratification of APE. Therefore, the aim of this study is to determine whether PAOI can be used as a simple quantitative index to assess the risk stratification of APE.

## Results

### CT manifestations of pulmonary embolism

**Figure 1:**
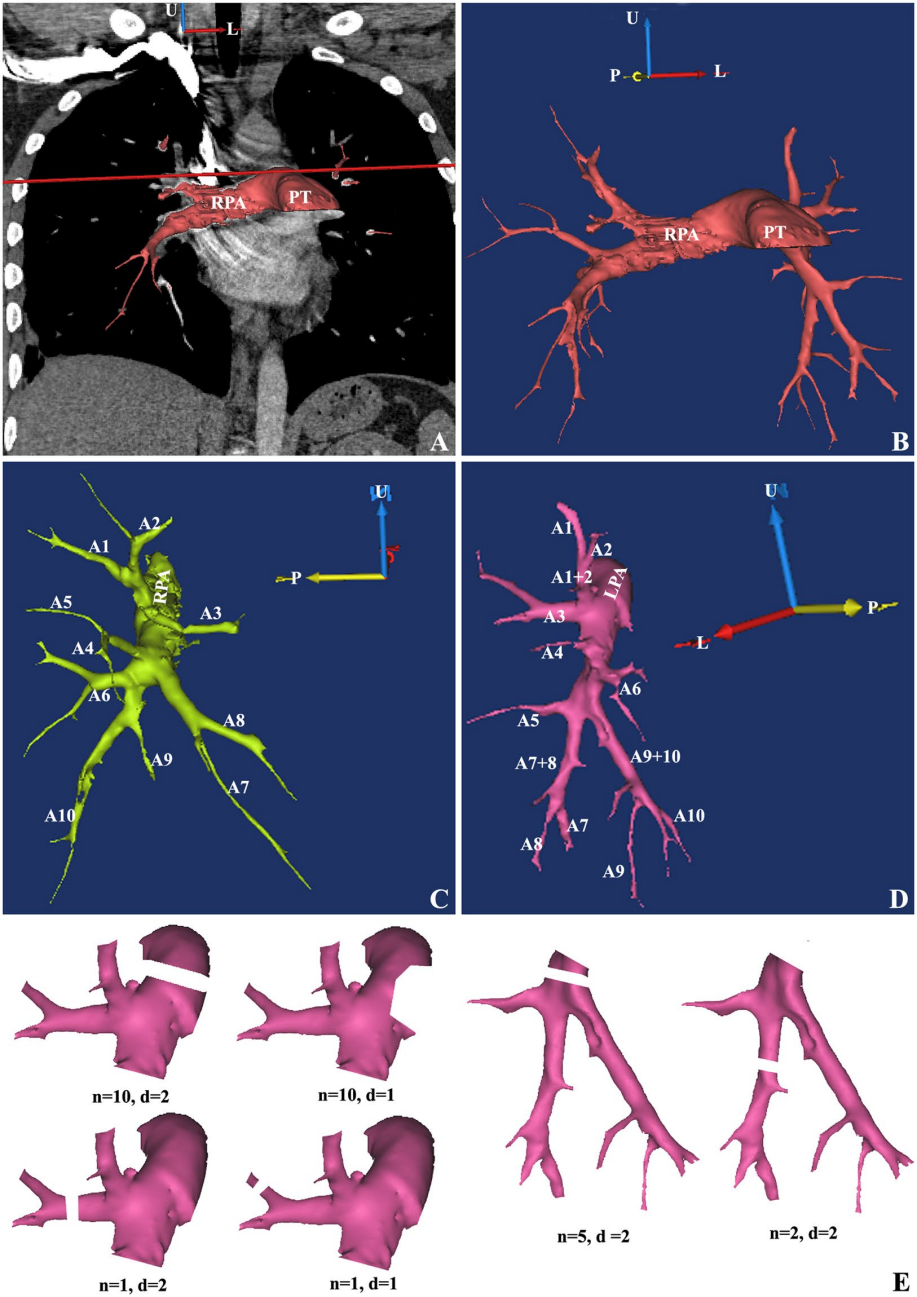
Analysis of branches of the pulmonary artery in a patient with APE. (**A**) The projection of branches of the pulmonary artery on coronal plane. (**B**) Holistic view of branches of the pulmonary artery. (**C**) Branches of the right pulmonary artery. (**D**) Branches of the left pulmonary artery. (**E**) Instruction of “*n*” and “*d*” in calculation formula of PAOI. U, up; L, left; P, posterior; PT, pulmonary trunk; RPA, right pulmonary artery; LPA, left pulmonary artery. A1, apical segmental artery; A2, posterior segmental artery; A3, anterior segmental artery; A4, lateral segmental artery (right side) or superior lingual segmental artery (left side); A5, medial segmental artery (right side) or inferior lingual segmental artery (left side); A6, superior segmental artery; A7, medial basilar segmental artery; A8, anterior basilar segmental artery; A9, lateral basilar segmental artery; A10, posterior basilar segmental artery.

The identification of the branches of pulmonary artery were showed in Fig. 1. The embolus in these branches had direct and indirect signs in CT images.

**Figure 2:**
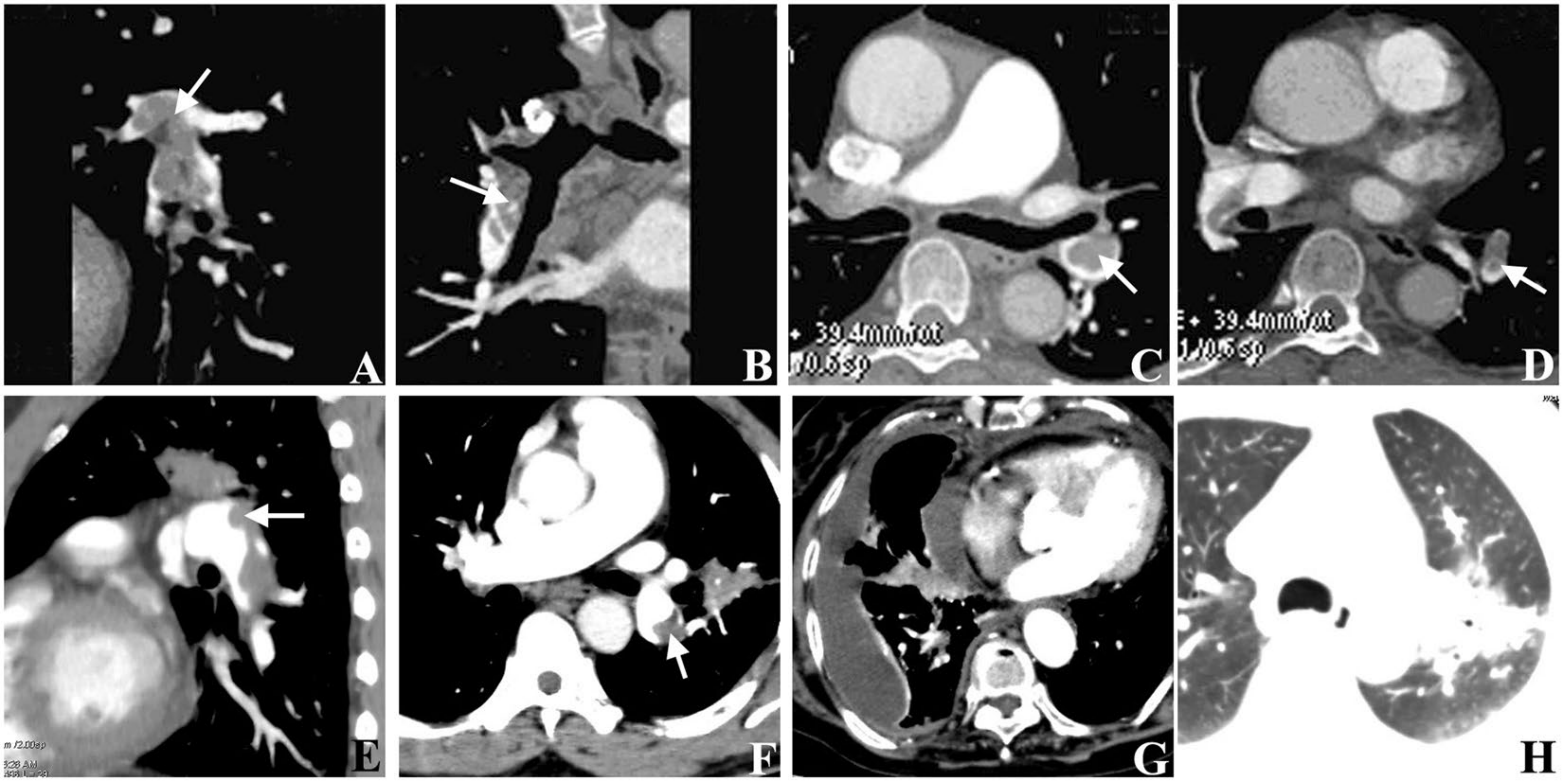
CT manifestations of pulmonary embolism. (**A**,**B**) The sagittal (**A**) and coronal (**B**) MPR images showed the right inferior pulmonary artery were filled with a filling defect, which belonged to a complete occlusion type of pulmonary embolism. (**C**,**D**) The original transverse axis images showed the central filling defect surrounded by contrast media in the left inferior pulmonary artery, which belonged to a central type of pulmonary embolism. (**E**,**F**) The coronal (**E**) and axis images (**F**) showed the filling defects in one side of the artery, which belonged to a peripheral type. (**G**) The pleural effusion and pericardial effusion was showed in one patient. (**H**) The pulmonary infarction was showed in one patient. Arrows indicate the embolus.

Direct signs: In combination with MPR, MIP and VR, the number and the location of the embolus were recorded. According to the position of embolus in the vessel lumen and the relationship between embolus and vessel wall, the emboluses were divided into three types: (1) The complete occlusion type was found in 42 vessels. MPR and MIP showed different filling defects in the lumen of arteries (Fig. 2A,B). VR showed stenosis or interruption of vascular lumen. (2) The central type was found in 27 vessels. Axial images and MPR images showed a low-density mass shadow in the lumen of the arteries. Contrast agents were present around the lesion. There were no significant changes in VR images. MIP showed the central density reduction area (Fig. 2C,D). (3) The peripheral type was found in 80 vessels. The original cross-sectional images, MPR and MIP images all showed filling defects in one side of the artery (Fig. 2E,F). The VR images showed that the diameter of the vessels in each segment was not equal.

Indirect signs (Fig. 2G,H): There were 9 cases of pleural effusion, 6 cases of pericardial effusion, 6 cases of pulmonary infarction, and 3 cases of mosaic sign.

### PAOI in patients with different risk stratification APE

**Figure 3:**
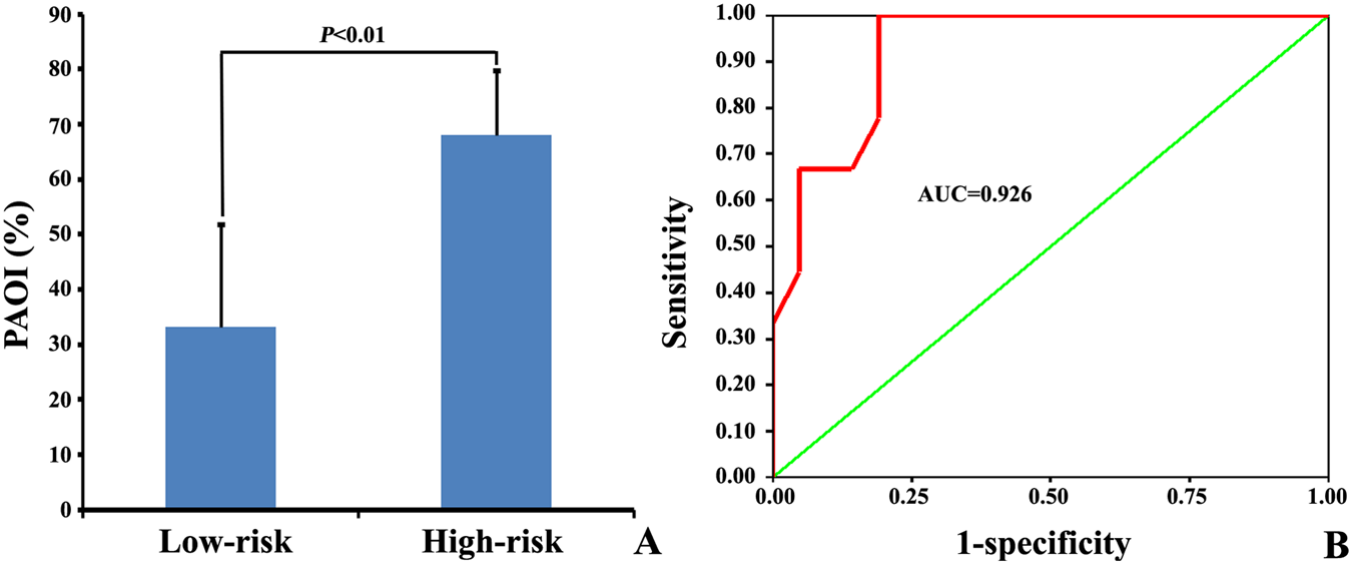
PAOI in different risk stratification APE patients. (**A**) Comparison of PAOI in low- and high-risk patients. (**B**) ROC curve of PAOI prediction for high-risk patients.

There were 21 cases of low-risk patients and 9 cases of high-risk patients. The PAOI in low- and high-risk patients was (33.2 ± 18.6)% and (68.1 ± 11.8)%, respectively (Fig. 3A), and the difference was statistically significant (*t* = 6.22, *P* < 0.01).

### Predictive effectiveness of the pulmonary embolism index for risk stratification

The ROC curve of PAOI prediction for high-risk patients was shown in Fig. 3B. Form the ROC curve, the cut-off value for the high-risk patients was 52.5%, the sensitivity was 100%, the specificity was 81.0%, the area under the curve was 0.926 (*P* < 0.01), the negative predictive value was 69.2%, and the positive predictive value was 100%.

### Cardiovascular parameters in patients with different risk stratification APE

**Figure 4:**
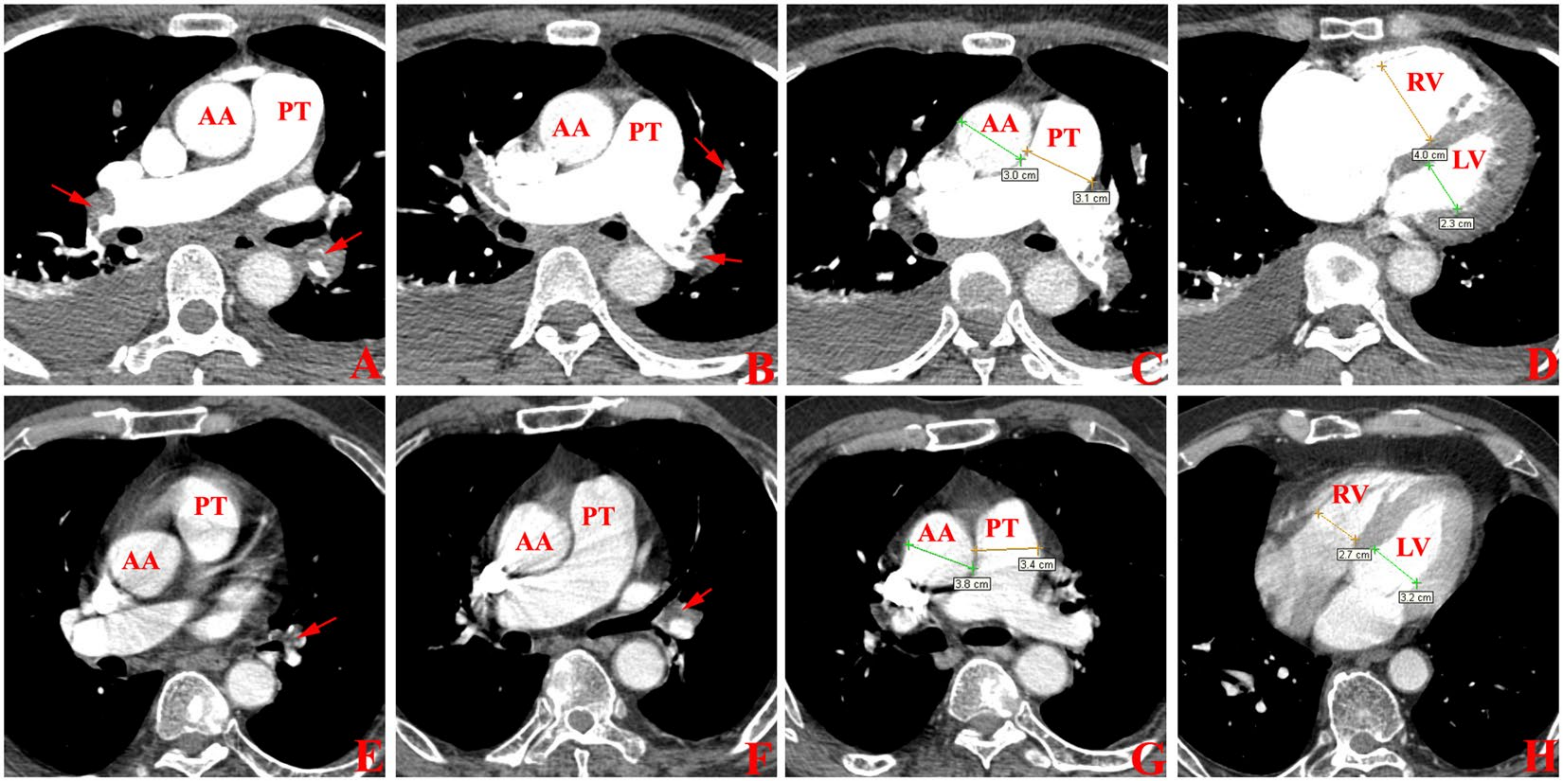
Cardiovascular parameters in low- and high-risk patients with APE. (**A**–**D**) One patient with high-risk APE. The complete occlusion was found in the right pulmonary artery and left inferior lobe artery. The PAOI was 75%, the rPA was 1.03, and the rRL was 1.74. (**E**–**H**) One patient with low-risk APE. The peripheral occlusion was found in the left inferior lobe artery. The PAOI was 12.5%, the rPA was 0.89, and the rRL was 0.84.

The cardiovascular parameters in low- and high-risk patients were shown in Table 1. Compared with low-risk patients, the PTa, rPA, RVa, rRL in high-risk patients were higher, and the LVa in high-risk patients was lower (Fig. 4). There was no statistical difference between the AAd between two groups.

**Table 1:** Cardiovascular parameters in patients with different risk stratification APE.

Groups	PTd(cm)	AAd(cm)	rPA	RVd(cm)	LVd(cm)	rRL
Low-risk	2.8 + 0.3 (2.2–3.4)	3.1 + 0.2 (2.9–3.2)	1.1 + 0.1 (1.0–1.3)	4.1 + 0.7 (3.1–5.2)	3.2 + 0.5 (2.1–3.8)	1.3 + 0.4 (1.0–2.1)
High-risk	3.5 + 0.4 (2.9–4.2)	3.1 + 0.2 (2.6–3.4)	0.9 + 0.1 (0.7–1.2)	3.5 + 0.5 (2.5–4.2)	4.0 + 0.7 (2.9–5.6)	0.9 + 0.1 (0.6–1.0)
*t* value	5.13	0.61	6.18	3.02	2.90	3.54
*P* value	<0.01	0.55	<0.01	0.01	0.01	0.01

Note: APE, acute pulmonary embolism; PTd, diameter of pulmonary trunk; AAd, diameter of ascending aorta; rPA, ratio of PTd to AAd; RVd, diameter of right ventricle; LVd, diameter of left ventricle; rRL, ratio of the RVd to LVd.

### Correlation between PAOI and cardiovascular parameters

The cardiovascular parameters MPAd, rPA, RVd and RVd/LVd were positively correlated with PAOI, and the correlation between RVd/ LVd and PAOI was the highest (r = 0.657, *P* < 0.05), LVd was negatively correlated with PAOI. The correlation with cardiovascular parameters was shown in Table 2.

**Table 2:** Correlation between PAOI and cardiovascular parameters.

Parameters	total	*r* value	*t* value
PTd (cm)	3.0 + 0.5 (2.2–4.2)	0.39	0.03
AAd (cm)	3.1 + 0.2 (2.6–3.4)	0.17	0.36
rPA	1.0 + 0.1 (0.7–1.3)	0.64	<0.01
RVd (cm)	3.7 + 0.6 (2.5–5.2)	0.58	<0.01
LVd (cm)	3.8 + 0.7 (2.1–3.6)	−0.44	0.02
rRL	1.0 + 0.3 (0.6–2.1)	0.65	<0.01

Note: PAOI, pulmonary artery obstruction index; PTd, diameter of pulmonary trunk; AAd, diameter of ascending aorta; rPA, ratio of PTd to AAd; RVd, diameter of right ventricle; LVd, diameter of left ventricle; rRL, ratio of the RVd to LVd.

## Discussion

Patients with APE lack specific clinical symptoms and are easily misdiagnosed with other diseases^19^. It is reported that more than 95% of APE patients are characterized by dyspnea and chest pain, which are easily misdiagnosed by clinicians as acute coronary syndromes and lung diseases at the beginning of the disease, and even increase the fatality rate of these type of patients^20,21^. According to statistics, pulmonary embolism is the third most common cause of death in Western developed countries^22^. Therefore, patients with clinically suspected APE should be diagnosed as soon as possible, and the severity of disease should be assessed at the same time to help selecting treatment strategies^23^. Hypotension, shock, syncope and cardiac arrest are all high-risk signs of APE, and prompt treatment is needed^24^. The ESC 2008 APE guidelines emphasize that the severity of APE should be based on an individualized assessment of the early death risk associated with APE^11^. The new 2014 APE guide adds new indicators to further refine the risk stratification, including the patient’s age, sex, underlying disease, clinical symptoms, and many other indicators sometimes observed in practical clinical work^12^. However, it is difficult to assess the condition of APE patients in a timely manner.

With continued development of diagnostic technology (especially with the appearance of multi-detector and dual-source CT in recent years), thin-layer scanning and thin-layer reconstruction now possess greatly improved image resolution, powerful post-processing function and have the ability to collect large quantities of information^25–28^. It can clearly display the main pulmonary artery and its 5~6 branches, and can be observed from any angle and direction^29^. The pulmonary artery can directly display the relationship between the whole lung blood vessel and the embolic space structure, and has greatly enhanced the evaluation of the severity of pulmonary embolism^30^. Ghaye *et al*. proposed that CTPA can replace echocardiography in assessing the “gold standard” status of right ventricular dysfunction, because it can effectively measure APE, and at the same time measure PAOI and cardiovascular structure to quantify the degree of pulmonary embolism and right heart function while making a quantitative diagnosis^31^. At present, the PAOI was commonly used by the Mastora embolism index^32^ and Qanadli embolism index methods^17^. The evaluation method of the Qanadli embolization index is widely used for its simple and easy clinical application.

In 2009, 85 patients with suspected APE were examined by CTPA, of whom 53 were diagnosed with PE. The patients were divided into the hemodynamic instability group (*n* = 20) and the hemodynamic stability group (*n* = 33) according to their hemodynamic status. There were significant differences in the PAOI. of two groups. ROC analysis revealed that the threshold value of PAOI for diagnosing hemodynamic instability was 48% (sensitivity 95% and specificity 76%)^33^. In 2010, some of the 73 patients with APE were divided into critical and non-critical restructures according to the clinical indicators. It was found that the PAOI was significantly higher than the non-critical recombination (median 43% vs 20%), and was related to the right cardiac insufficiency^34^. In 2012, researchers found that in the death and survival groups of patients with acute pulmonary embolism, an increase in PAOI was a risk factor for death in patients with acute pulmonary embolism. The negative predictive value of death reached 100% when the PAOI was less than 40%^35^.

No correlation has been found between the PAOI value and the risk stratification of APE patients. The PAOI method is simple, with low equipment requirements and can be accurately evaluated without special software. It can be widely used in clinical practice. In this study, the PAOI value and risk stratification of 30 patients with APE were compared with the risk stratification. It was found that there were significant differences in the PAOI value between the patients with low-risk and high-risk stratification, and the higher PAOI (>52.5%) predicts the patients with high risk, for which the sensitivity and specificity was 100% and 81.0%, respectively. Therefore, for APE patients, the higher the PAOI value, the higher the risk stratification is. As the risk stratification is closely related to the early mortality of patients, the measurement of PAOI has important reference value for clinical assessment of APE patients.

This study further observed the correlation between PAOI and other cardiovascular parameters. The results showed that there was a correlation between PAOI and cardiovascular parameters. The correlation between PAOI and RVd/LVd was the highest, which was consistent with the previous study, suggesting that PAOI could prodict the severity of hemodynamic changes in APE patients. After the occurrence of APE, the embolus can cause pulmonary artery contraction through mechanical obstruction and nerve body fluid regulation and hypoxia, which leads to increased pulmonary circulation resistance and increased pulmonary arterial pressure in different degrees. The right ventricular and pulmonary vascular post load increases significantly when the patients are in a seriously compromised cardiovascular state, causing right ventricular and pulmonary vascular decompensation adjustment, even in the right ventricular and pulmonary vessels, and secondary acute right heart failure. Decompensation regulation mainly manifested as the dilatation of the right ventricle, the widening of the main trunk of the pulmonary artery, the localized dilatation of the upstream vessels of the embolic part and the compensatory expansion of the un-embolized pulmonary vessels. The main manifestations were a decrease of blood pressure and an increased likelihood of shock. The results of this study show that there were different levels of correlation between PAOI and cardiovascular parameters in APE patients. With the increase of PAOI, the pulmonary artery pressure increased and the cardiovascular parameters changed to varying degrees. The right ventricular function was most obvious. This maybe the reason why PAOI can predict high-risk patients.

## Conclusion

Although DSA pulmonary angiography is the gold standard for the diagnosis of pulmonary embolism, CTPA is the first choice for acute pulmonary embolism in emergency patients. CTPA has the advantages of being non-traumatic, capable of rapid imaging, high accuracy, and the production of a clear image. The distribution range and size can be used for three-dimensional imaging. Sem-quantitative analysis of PAOI is helpful for risk stratification in patients with acute pulmonary embolism, and it can be used to guide the treatment of patients. A limitation of this investigation is the small number of cases that were available to be included in the study. In the future, we will consider the application of PAOI in guiding the treatment of patients.

## Materials and Methods

### Research object

The subjects of this study were the patients with APE admitted to the First Affiliated Hospital of Bengbu Medical College. Inclusion criteria were as follows: (1) there was visible arterial embolization in a CT image, (2) time of onset within 14 days, and (3) no thrombolytic or anticoagulant therapy was carried out before admission.

Exclusion criteria were as follows: (1) chronic pulmonary hypertension or right ventricular wall thickening etc., (2) congenital heart disease, (3) coronary heart disease, (4) abnormal left heart function, (5) pulmonary and mediastinal diseases that may affect the diameter of pulmonary artery, and (6) poor image quality, such that the reconstruction could not be completed. A total of 30 cases were selected, including 14 males and 16 females, aged (64.9 ± 15.9) years.

The study was approved by the Ethics Committee of The First Affiliated Hospital of Bengbu Medical College. All the participants provided their written informed consent to participate in this study.

All methods were performed in accordance with the relevant guidelines and regulations.

### CT scanning methods

A General Electric (GE) Lightspeed 64-row spiral CT was used. The scanning range was selected from the thoracic inlet to the costophrenic angle in the supine position. The voltage and current of scanning parameters are 100 kV and 50 mA respectively. The layer thickness was set to 0.625 mm. A double cylinder high-pressure syringe was used for intravenous injection. The injection dose of Ioversol (I) (370 mgI/ml) was calculated according to the weight of 1 ml/kg, while the injection speed was maintained at rate of 4–5 ml/s and a double flow rate was maintained throughout. The scan started after the contrast agent was injected for 18–28 s. The original data were analysed by three-dimensional post-processing techniques such as multi-planner reformation (MPR), maximum intensity projection (MIP), and volume rendering (VR), to obtain the corresponding embolic locations, numbers, and morphological features. All the CT images were reviewed by two radiologists, independently. If there was any inconsistency between them, another board-certified radiologist participated in discussion until the results agreed with each other.

### Calculation of PAOI

The calculation formula for PAOI was as follows:

1 $$PAOI=[\frac{\sum (n\times d)}{40}]\times 100 \% $$

In the formula, the “*n*” represents the embolus score for a special pulmonary segment. Each lung is divided into 10 pulmonary segments, and each segment had a main pulmonary segmental artery. The names of all arteries are shown in Fig. 1.

Three computational method were used (Fig. 1E). Firstly, if the embolus was found in one pulmonary segmental artery, a score of 1 was assigned. Secondly, if the embolus was found in the sub-segmental pulmonary artery, the score was also 1 and was considered to be the result of partial obstruction of the corresponding pulmonary segmental artery. Finally, if the embolus was found in the directional branch of the left or right pulmonary artery (that is, above the pulmonary segmental artery), the score was the total number for the pulmonary segmental artery. Thus, the range of “*n*” was from a minimum of 1 (a blockage of a pulmonary segmental artery) to a maximum of 20 (bilateral pulmonary artery occlusion).

In the formula, “*d*” represents the degree of thrombus for each artery, with a partial obstruction of 1 and a complete occlusion of 2 (Fig. 1E). The final result of PAOI is expressed as a percentage.

### Measurement of cardiovascular parameters

Measurement of the diameter of pulmonary trunk and ascending aorta was carried out as follows: On the horizontal axis image in CTA, the measuring line was drawn at the widest area of pulmonary trunk, perpendicular to the long axis of the artery. The maximum diameter of pulmonary trunk (PTd) was measured. The diameter of ascending aorta (AAd) was measured at the same plane. The ratio of the diameter of pulmonary trunk to the diameter of aorta (rPA) was then calculated, and the calculation formula was as follows:

2 $$rPA=\frac{PTd}{AAd}$$

Measurement of the maximum diameter of the short axis of left ventricle and right ventricle was as follows: On the horizontal axis image in CTA, the measuring line was drawn at the widest area of left ventricle and right ventricle. The maximum distances between the ventricular septum and the free wall of left and right ventricular were measured, which was defined as the diameter of left ventricle (LVd) and the diameter of right ventricle (RVd). The ratio of the RVd to LVd was then calculated (rRL), and the calculation formula is as follows:

3 $$rRL=\frac{RVd}{LVd}$$

### Definition of risk stratification

The risk stratification was performed with reference to ESC 2008 APE guidelines. (1) Shock or hypotension refers to the systolic pressure of the body circulation <90 mmHg or a decrease of the base value over 40 mmHg that lasts for more than 15 minutes. Those patients whose hypotension was caused by new arrhythmia, low blood volume, or infection were excluded. (2) Right ventricular dysfunction (RVD) refers to the observation of at least one of the following criteria: cardiac colour Doppler ultrasound indicated right ventricular systolic dysfunction, CT showed right ventricular enlargement, brain natriuretic peptide (BNP) elevation (>90 ng/L), NT-BNP increase (>500 ng/L), electrocardiogram (ECG) showed new onset complete or incomplete right bundle branch block, and ST segment elevation or depression or T wave inversion. (3) Myocardial injury: cardiac troponin I > 0.4 µg/L or troponin T > 0.1 µg/L.

Patients with no right ventricular dysfunction and myocardial injury markers were classified as low-risk group, while patients with shock or persistent hypotension were classified as high-risk group.

### Statistical method

Statistical processing was performed by using the SPSS 21 software package. Measured data in accordance with the normal distribution were expressed as mean ± standard deviation. Independent quantitative t-tests were used to compare the quantitative data between the groups, and a χ^2^ test was used for the qualitative data. The correlation between PAOI and cardiovascular parameters was analysed using Spearman correlation analysis. The maximum likelihood method for PAOI was used to fit the receiver operating characteristic curve (ROC) to the low-risk group and high-risk group, and the area under the curve (AUC) was calculated. *P* < 0.05 was considered statistically significant.

## Data Availability

All data generated or analyzed during this study are included in this published article. The original data also can available from the corresponding author on the reasonable request.
